# Angiogenesis imaging study using interim [^18^F] RGD-K5 PET/CT in patients with lymphoma undergoing chemotherapy: preliminary evidence

**DOI:** 10.1186/s13550-021-00776-9

**Published:** 2021-04-12

**Authors:** David Tonnelet, M. D. Pierre Bohn, Stephanie Becker, Pierre Decazes, Vincent Camus, Sebastien Thureau, Hervé Tilly, Fabrice Jardin, Pierre Vera

**Affiliations:** 1 Department of Nuclear Medicine, Henri Becquerel Cancer Center, Rouen University Hospital & QuantIF-LITIS, University of Rouen, 1 rue d’amiens, 76000 Rouen, France; 2 Inserm U1245 and Department of Hematology, Henri Becquerel Cancer Center, Rouen University Hospital & QuantIF-LITIS, University of Rouen, Rouen, France; 3 Depatment of Radiotherapy Henri Becquerel Cancer Center, Rouen University Hospital & QuantIF-LITIS, University of Rouen, Rouen, France

**Keywords:** RGD, K5, FDG, PET/CT, Lymphoma, Angiogenesis

## Abstract

**Background:**

Our aim was to measure the impact of two cycles of standard chemotherapy on tumoural neoangiogenesis by [^18^F] fluorine arginine-glycine-aspartic (RGD-K5) positron emission tomography–computed tomography (PET) on patients presenting with lymphoma. Nineteen patients at Rouen’s Henri Becquerel Cancer Centre were prospectively included. Fluorodeoxyglucose (FDG) and RGD-K5 PET were performed before (C0) and after (C2) two cycles of chemotherapy. End-of-treatment FDG PET was performed for final evaluation. Maximum standardised uptake value (SUVmax), SUVmean, Metabolic Tumour Volume (MTV) and Angiogenic Tumour Volume (ATV) were measured for all lesions. RGD SUVmax and SUVmean were also analysed in 13 normal organs at C0 and C2. The patient’s treatment response was considered using the Deauville score (DS) at the end of FDG PET treatment (DS 1–3 were considered responders, and 4 and 5 non-responders).

**Results:**

Eighteen patients had both C0 FDG and RGD PET. Twelve patients had both C2 FDG and RGD, completed the treatment protocol and were included in end-of-treatment analysis. No statistical difference was found in RGD uptake of normal organs before and after chemotherapy for SUVmax and SUVmean. On C0 RGD, apart from classical Hodgkin lymphoma (cHL; *n* = 5) and grey zone lymphoma (GZL; *n* = 1), other lymphoma sub-types (*n* = 12) had low RGD uptake (*p* < 0.001). Regarding FDG, there was no significant difference for SUVmax, SUVmean and MTV at C0 and C2 between patients with cHL and non-Hodgkin lymphoma (NHL). At C2 RGD, non-responders had higher SUVmax and SUVmean compared to responders (*p* < 0.001). There was no significant difference in RGD ATV between responders and non-responders.

**Conclusions:**

Our study showed significant higher initial RGD uptake in patients presenting with cHL and GZL compared to NHL. Non-responder also had higher post-chemotherapy RGD uptake compared to responders. Issues raised by RGD uptake, particularly in cHL, are yet to be explored and need to be confirmed in a larger population.

**Supplementary Information:**

The online version contains supplementary material available at 10.1186/s13550-021-00776-9.

## Background

Angiogenesis is a fundamental process involved in a variety of physiological and pathological conditions. The growth of solid tumours remains restricted to 2–3 mm in diameter until the onset of angiogenesis [1]. The concept of antiangiogenic therapy in clinical oncology is to stop cancer progression by suppressing the tumour’s blood supply, and more than 20 angiogenic growth factors have been studied, including their receptors and signal transduction pathways. This increasing use of targeted therapies has led to a growing demand for imaging the tumour response to these treatments. Such biomarkers not only would facilitate clinical trials of new drugs but could also be used to aid in the selection of optimal treatment for individual patients (personalised medicine).

Positron emission tomography–computed tomography (PET) using tracers for the assessment of glucose metabolism by [^18^F] fluorodeoxyglucose (FDG) is well established, and it plays a crucial role in initial staging and treatment response assessment, especially in patients with lymphoma [2]. Integrin αvβ3 is a heterodimeric transmembrane glycoprotein consisting of α- and β-subunits which plays an important role in angiogenesis [3]. These glycoproteins interact in cell–cell and cell–matrix interactions, easing endothelial growth and cell migration, therefore paving the way to angiogenesis [4]. Integrins assist in the progress of tumour development and metastasis by facilitating endothelial and tumour cell migration. An important phenomenon that depends on cell–extracellular matrix (ECM) interactions is the growth or sprouting of new blood vessels from a pre-existing vascular bed [4]. It has been found that several ECM proteins, like vitronectin, fibrinogen and fibronectin, interact with integrins via the amino acid sequence arginine–glycine–aspartic acid (RGD in the single letter code) [3]. Based on these findings, monomeric, multimeric and cyclic peptides, including the RGD sequence have been introduced to allow integrin αvβ3 imaging and picture pathological angiogenesis. Targeting specific angiogenesis molecular markers by PET imaging, like the integrin αvβ3, can be used for angiogenesis imaging. Li et al. recently showed that high RGD uptake on pre-treatment PET predicted antiangiogenic response in refractory patients presenting with solid cancer [5]. Patients presenting with solid tumours that showed low RGD uptake did not benefit from the antiangiogenic effect. Patients presenting with high RGD uptake benefited from the antiangiogenic effect. Therefore, RGD imaging could also be used for response assessment of antiangiogenic therapies.

[^18^F] fluorine arginine-glycine-aspartic (RGD-K5) PET/CT has been used in clinical studies on patients presenting with head and neck cancer [6], breast cancer [7] and lung cancer [8]. To the best of our knowledge, no clinical imaging study of angiogenesis in patients presenting with lymphoma has been performed.

The main objective of this study was to measure the impact of two cycles of standard chemotherapy on tumoural neoangiogenesis assessed by RGD-K5 PET/CT in patients presenting with lymphoma. The secondary objective was to analyse RGD uptake according to the lymphoma subtype.

## Methods

### Population and treatment

This prospective study included 21 patients at Rouen’s Henri Becquerel Cancer Centre who presented with biopsy-proven lymphoma between July 2016 and November 2019 (Table 1). To avoid a partial volume effect on RGD PET, only patients with a target lesion at least 3 cm in size on CT were included. All patients were treated according to standard routine practice and national guidelines and their histological subtype. Patients with indolent lymphomas and no treatment criteria did not receive treatment. All patients were staged using FDG PET/CT. Additional RGD-K5 PET/CT was performed before or after initial and interim FDG (median + 7 days, range –4; + 22 for initial FDG and median –0.5 days, range –5; + 1 for interim FDG). Interim imaging using FDG and RGD was performed after 2 cycles of chemotherapy for patients receiving treatment. No additional treatment was given between FDG and RGD imaging. All patients had a final FDG for final Deauville score evaluation at the end of treatment. Patients with a final Deauville score of 4 or 5 were considered non-responders and those with score of 1, 2 or 3 as responders [9]. No end-of-treatment RGD PET was performed. All participants were followed at least 12 months after the end of treatment. The study was conducted in accordance with the precepts of the Helsinki Declaration and received approval by the Ethical Committee. All patients gave written consent for the study. A favourable opinion from the North-west Committee for the Protection of Persons was given (ref. CPP 02/008/2014). The EudraCT number is 2015–000757-20 and the study’s National Clinical Trial identifier is NCT02490891, first posted on 7 July 2015 (https://www.clinicaltrials.gov/ct2/show/NCT02490891).

**Table 1:** Baseline characteristics of included patients

Clinical features	Value
Mean age at diagnosis ± SD (years)	55 ± 13
Mean height ± SD (cm)	171.6 ± 7.55
Mean weight ± SD (kg)	78 ± 16
Sex (n)	
Female	7
Male	12
ECOG performance status (n)	
0	11
1	7
2	1
3–4	0
Histological subtype (n)	
Classical Hodgkin lymphoma	6
DLBCL	4
Follicular lymphoma	3
Mantle-cell lymphoma	3
T-cell lymphoma	1
Poppema	1
Grey zone lymphoma	1
Tumour stage (n)	
IE	1
II A	3
II B	4
III	2
IV	9
Bone marrow biopsy involvement (n)	
Negative	13
Positive	5
Unknown	1
Nodal site (n)	18
Extra-nodular site (n)	
Spleen	3
Lung	1
Liver	1
Skin	1
Tongue	1
Mean LDH level ± SD (UI/L)	485 ± 256
Chemotherapy type (n)	
ABVD	7
R-CHOP	6
None	3
Bendamustine	1
R-CVP	1
ACVBP	1
Prophylactic intrathecal methotrexate	
Yes	2
No	17
Number of patients analysed (n)	19
Total number of patients (n)	21

### PET-CT imaging

Patients underwent baseline (C0) and interim (C2) FDG and RGD PET. Regarding the protocol, interim RGD was not performed at C2 if it was negative at C0 (RGD was considered negative if SUVmax was ≤ 1 at C0). FDG was performed according to European Association of Nuclear Medicine (EANM) procedure guidelines [10]. For this procedure, 3.5 MBq/kg of [^18^F] FDG was injected after 20 min of rest. Sixty minutes later (± 10 min), the acquisition began with non-injected CT (100 kV; 80 mAs) in the cephalocaudal direction on a General Electric 710 PET/CT (Buc, France). The images were acquired with the patient’s arms positioned over the head while breathing freely. The PET data were then acquired in the caudocephalic direction using a whole-body protocol (2 min per bed position). The delay between injection and acquisition was 60 min.

^18^F RGD was provided by Siemens® PETNET. Production, labelling, delivering and Quality Control results were performed and provided by Siemens® PETNET. (Radiochemical purity > 95%, radiochemical impurities are in trace amount, i.e. of the order of less than 1% of the total radioactivity. The radiopharmaceutical preparations of RGD-K5, intended for parenteral administration, has been sterilised by a terminal sterilisation using a 0.22µ filter according to a validated industrial process. Patients did not fast prior to RGD administration. A standard dose of 4 MBq/kg (maximum 450 MBq) was administered after 60 min of rest. Images were acquired on the same PET instrument as for FDG, using the same bed protocol, but with parameters of 100 kV and 80 mAs CT to avoid unnecessary irradiation. The estimated additional dose delivered for RGD was 10 mSv. The tracer uptake was quantified using the standardised uptake value (SUV), calculated as tissue concentration (Bq/g)/[injected dose (Bq)/body weight (g)].

### PET analysis

All FDG and RGD PET scans were read by 2 nuclear physicians, one junior (2 years’ experience) and one senior (over 20 years’ experience). On FDG, all lesions (primary tumour and involved lymph nodes) with significant uptake were considered for determining maximal standardised uptake value (SUVmax), mean SUV (SUVmean) and metabolic tumour volume (MTV) using a 41% of SUVmax threshold.

On RGD, in addition to SUVmax, SUVmean for all lesions, SUVmax and SUVmean were also considered for 13 regions: occipital cortex, thyroid, ascending aorta (wall not included), inside left ventricle, lung, liver, spleen, gallbladder, kidney cortex, iliopsoas muscle, femoral head, T12 vertebra and inferior vena cava. All measurements were made on the right side (when applicable) regarding the middle third of the organ. For the liver, measurements assessed by placing a spherical volume of interest (VOI) of diameter 3 cm in the right upper lobe of the liver, avoiding the edge and any single ‘hot’ pixels likely to represent noise, sampling several axial slices to obtain a representative maximum liver SUVmax and SUVmean[10] (Additional file 1: Figure). For the spleen, measurements were made in the middle third. Organs were excluded from normal uptake analysis if they were found to be pathological on FDG PET or CT. For RGD, we measured an Angiogenic Tumour Volume (ATV) which mirrors the MTV only for RGD not FDG. The threshold for the ATV measurement was determined by the adaptive method and, if necessary, by expert visual sampling, i.e. experts visually adapted the threshold for the ATV to coincide with the tumour RGD uptake.

### Treatment and follow-up

All patients were treated according to national guidelines based on their histological subtype. Chemotherapy was performed using standard protocols. The patients were followed up by physical examination and FDG imaging. Biopsy was performed for any suspicious residual/recurrent tumours whenever possible.

### Histopathological analysis

All patients had biopsy-proven lymphoma. All tumour tissues were routinely fixed in 4% buffered formaldehyde and processed by standard methods into paraffin blocks, and 4-µm slides were prepared and stained with haematoxylin and eosin. A senior pathologist noted the number of vascular sections and the presence of capillary vascularisation via ERGmarker by field of view. For the ERG antibody (EP111, 1/100, pH 9; Epitomics, Burlingame, CA, USA), immunochemistry was performed with VECTASTAIN ABC Rabbit IgG Kit (Vector Laboratories, Burlingame, CA, USA). Samples were examined under an Olympus DX51 microscope (Olympus, Paris, France). All pathological samples were reviewed by one senior pathologist.

### Statistical analysis

Mean and standard deviation were used for descriptive data. Student’s t-test (or Mann-Whiney test for population sample < 5) was performed for patients and PET comparison. All significance thresholds were set at 0.05 (2-tailed test). Statistics were analysed using MedCalc® software (version 13.1.2; MedCalc Software bvba, Ostend, Belgium).

## Results

The patients’ clinical data are summarised in Table 1. In all, 19 patients (7 women and 12 men) were included. Mean age at diagnosis was 55 ± 13 years. ECOG performance status was ≤ 1 for 18 patients (95%; ECOG performance status = 2 for the remaining patient). Nine patients had stage IV lymphoma (47%). The median follow-up time was 17.8 months (range 12–28). No patient died during or after treatment. One patient had synchronous follicular and classical Hodgkin lymphoma, thus both primary lesions were analysed. All 19 patients had both C0 FDG and RGD PET; 12 patients had both C2 FDG and RGD, completed the treatment protocol and were included for end-of-treatment analysis.

**Fig. 1:**
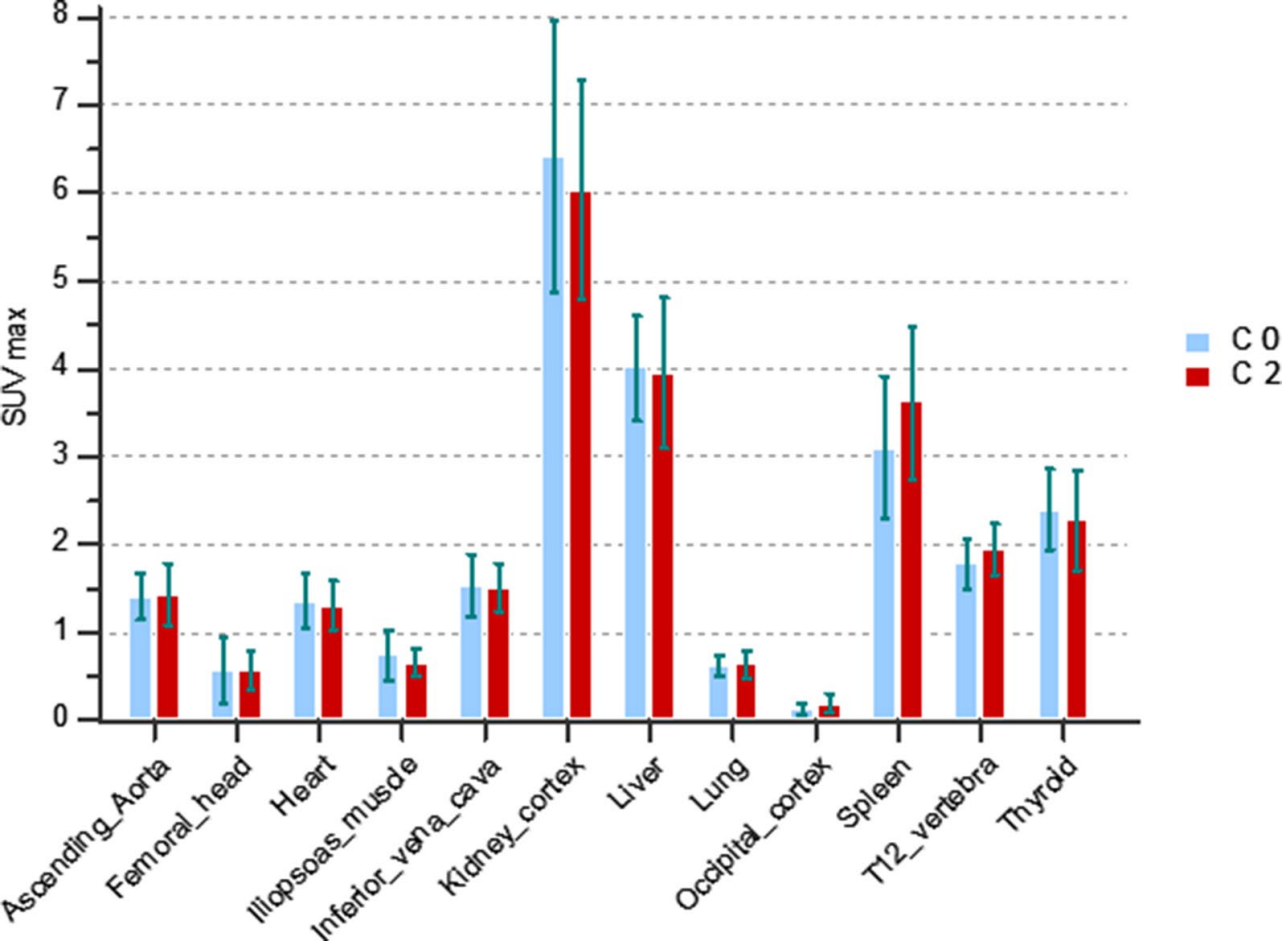
SUV max uptake in organs before (C0) and after (C2) chemotherapy.

SUVmax for normal RGD organs is shown in Fig. 1 No statistical difference was found in normal organs before (C0) and after (C2) chemotherapy for SUVmax and SUVmean. High RGD uptake in the gallbladder (SUVmax 15.5 ± 6.7 (range 6.8–25.8) on C0 and 18.4 ± 9.6 (range 6.3–34.7) on C2, not shown in Fig. 1 for ease of visualisation), was due to radiotracer elimination.

Ex vivo analysis was performed on the initial biopsies of 10 patients. No correlation was found between endothelial cell marking via the ERGmarker, the number of vascular sections and C0 and C2 RGD uptake (data not shown).

**Fig. 2:**
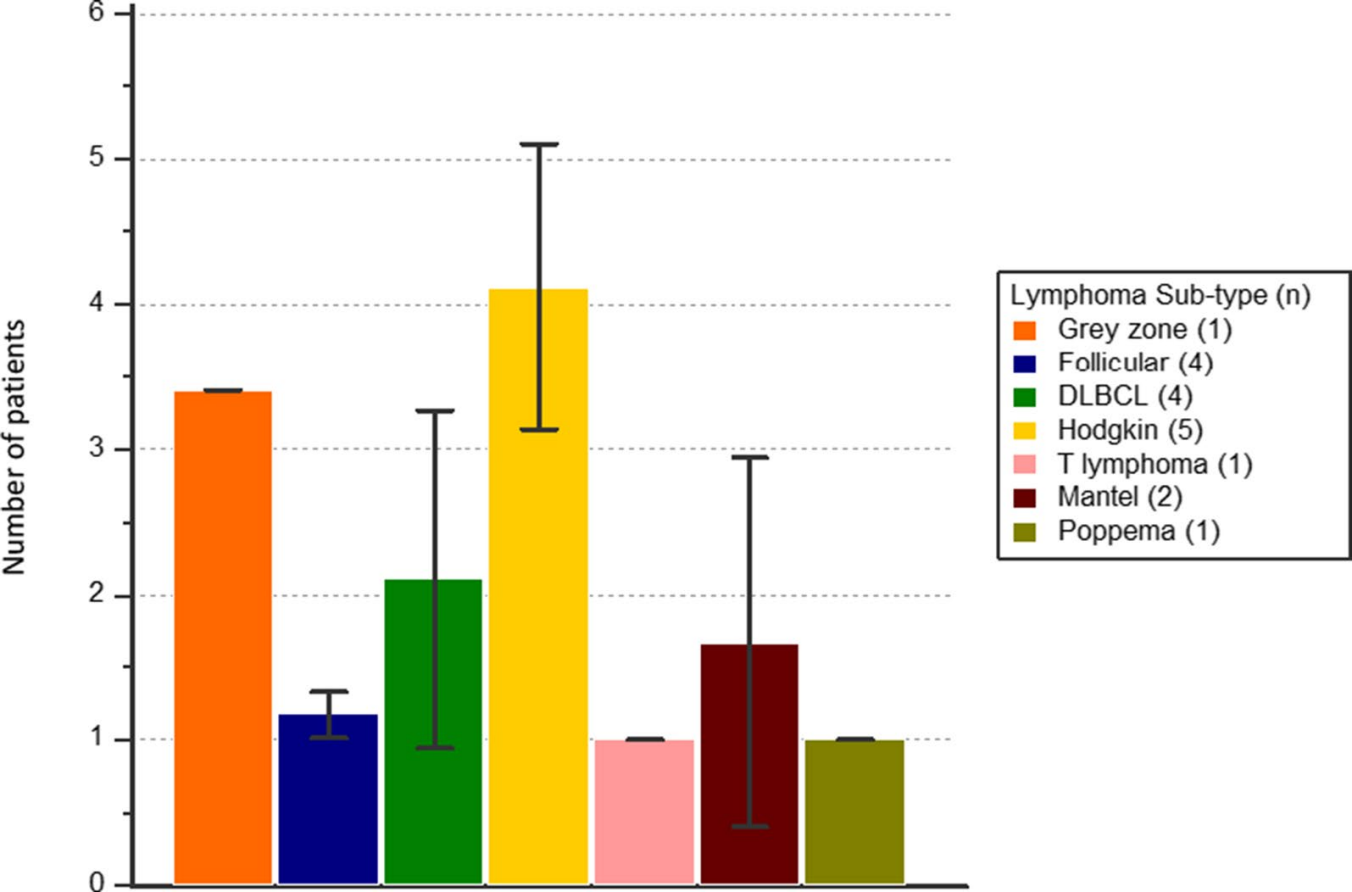
C0 RGD uptake according to histological subtype

RGD uptake was analysed according to histological subtype (Fig. 2). Apart from classical Hodgkin lymphoma (HL) and grey zone lymphoma (GZL), other lymphoma subtypes had low RGD uptake. RGD uptake was therefore compared between cHL and non-Hodgkin lymphoma (NHL), including grey zone lymphoma.

**Fig. 3:**
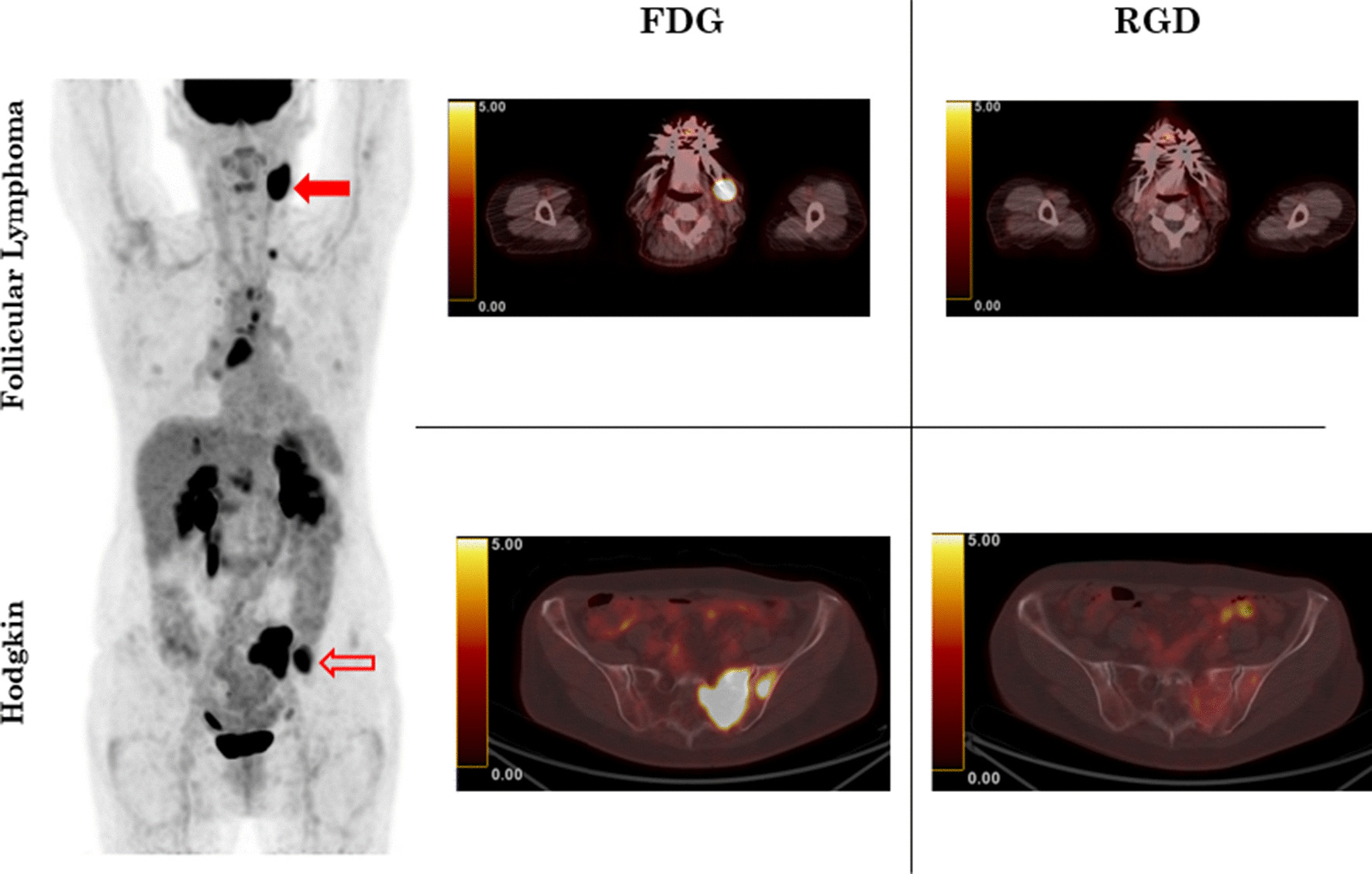
FDG maximum intensity projection (left) in patient presenting with synchronous biopsy-proven follicular (full red cross) and Hodgkin (empty red cross) lymphoma. On axial PET/CT (middle and right column), both lesions had significant FDG uptake (middle column). No RGD uptake in follicular lymphoma (right column, upper row). Significant RGD uptake in Hodgkin lymphoma (right column, bottom row)

To illustrate these results, Fig. 3 shows information on a patient who had synchronous follicular and classical Hodgkin lymphoma. The patient had a history of cHL in 2014 which was considered in full remission after eight ABVD cycles, with early bone relapse in 2015. Bone relapse was successfully treated with four ICE cycles. The patient could not undergo autologous bone marrow transplant due to stem cell collection failure. In September 2017, the patient saw her haematologist for cervical swelling. Cervical biopsy revealed grade II follicular lymphoma. Initial FDG revealed lymph node and left sacral wing involvement. However, RGD uptake was only significant for the left sacral wing. The dissimilarity between FDG and RGD uptake was questioned with regard to the patient’s histopathological subtype. Bone biopsy was performed and confirmed concomitant bone cHL relapse.

**Fig. 4:**
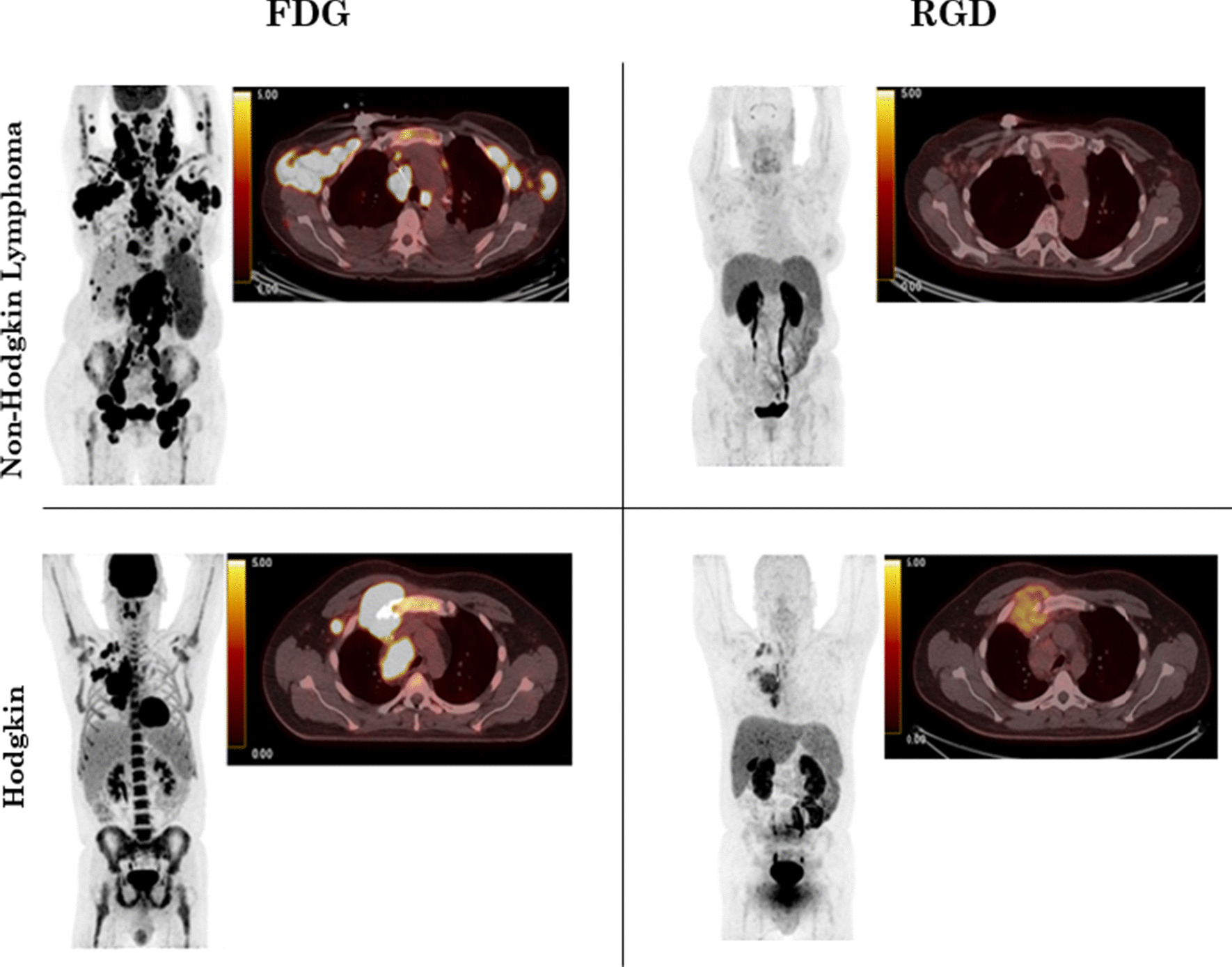
FDG (left column) and RGD (right column) uptake at baseline (C0) in two patients, one presenting with non-Hodgkin lymphoma (upper row) and one with classical Hodgkin lymphoma (bottom row)

Table 2 summarises FDG and RGD uptake at C0 and C2 for patients presenting with HL and NHL. At C0 and C2 FDG, there was no significant difference in SUVmax, SUVmean, MTV and ATV between patients with HL and NHL. At C0 RGD, patients with HL tended to have higher SUVmax, SUVmean and ATV than those with NHL. At C2 RGD, there was no significant difference in SUVmax, SUVmean and ATV between HL and NHL. An example is presented in Fig. 4.

**Table 2:** FDG and RGD uptake in Hodgkin and non-Hodgkin patients before (C0) and after (C2) chemotherapy

FDG	C0			C2		
	Hodgkin	Non-Hodgkin	*p*-value	Hodgkin	Non-Hodgkin	*p*-value
	*n* = 6	*n* = 13		*n* = 6	*n* = 10	
SUVmax ± SD	14.8 ± 4.7	17.0 ± 8.6	0.56	2.6 ± 1.3	5.1 ± 2.0	0.02
MTV ± SD	91.8 ± 60	254 ± 306	0.22	33 ± 65	27.6 ± 40.6	0.4
RGD	*n* = 6	*n* = 13		*n* = 5	*n* = 7	
SUVmax ± SD	3.9 ± 1.4	1.9 ± 1.1	0.002	3.1 ± 1.1	3.6 ± 1.8	0.6
ATV ± SD	145 ± 125	3.2 ± 9	< 0.001	46 ± 45	45 ± 99	0.9

**Fig. 5:**
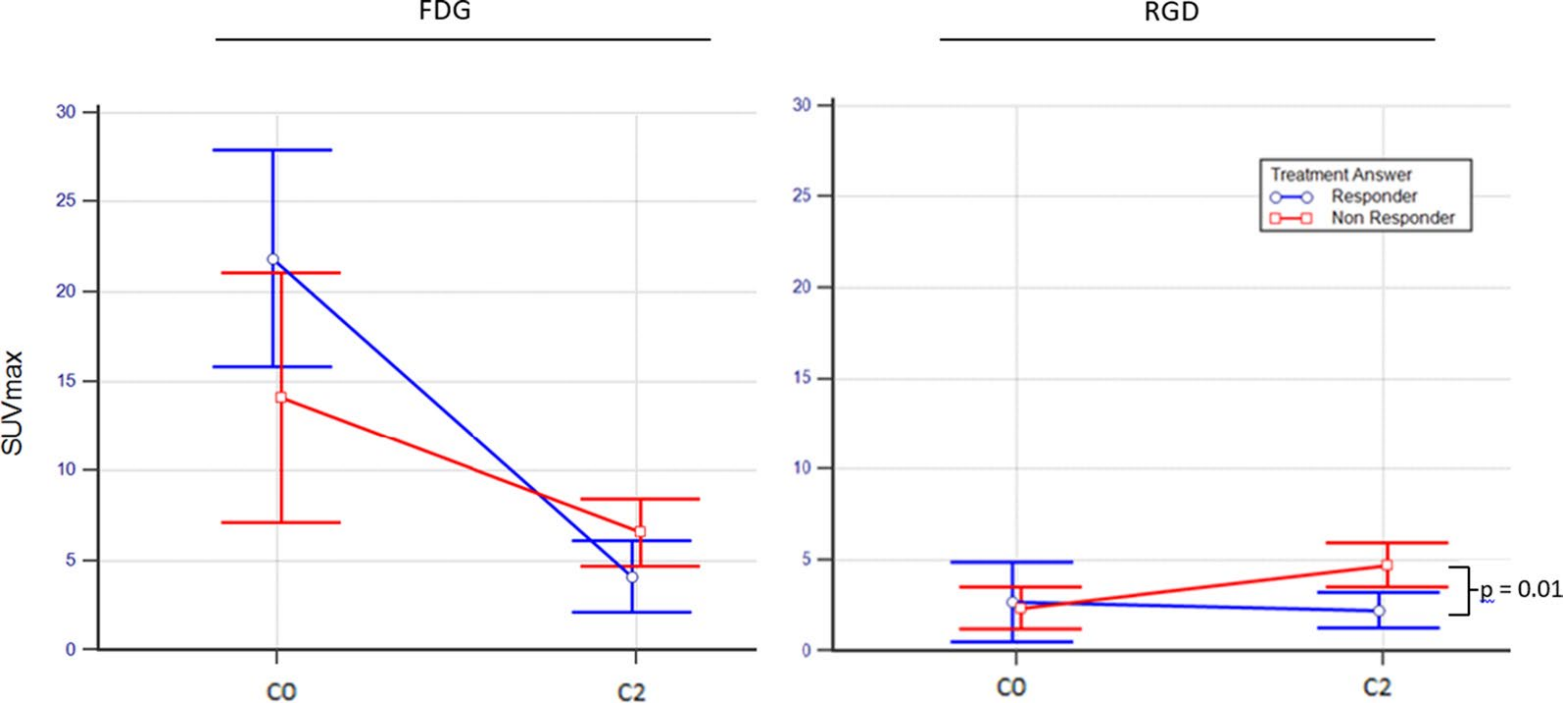
FDG (left) and RGD (right) SUVmax on initial (C0) and interim (C2) PET-CT according to final Deauville score (DS) (non-responder = 4 or 5, responder = 1, 2 or 3)

Table 3 summarises FDG and RGD uptake at C0 and C2 according to final Deauville score. At C0 and C2 FDG and C0 RGD, there was no significant difference in SUVmax, SUVmean, MTV and ATV between responders and non-responders. At C2 RGD, non-responders tended to have higher SUVmax and SUVmean compared to responder (Fig. 5). There was no significant difference in RGD ATV between patients who responded to treatment or not.

**Table 3:** FDG and RGD uptake on initial (C0) and interim (C2) PET-CT according to final Deauville score (DS) (responder = 4 or 5; non-responder = 1, 2 or 3)

FDG	C0			C2		
	Responder	Non-Responder	*p*-value	Responder	Non-Responder	*p*-value
	*n* = 6	*n* = 6		*n* = 6	*n* = 6	
SUVmax ± SD	18.3 ± 7.3	14.6 ± 5.7	0.35	3.4 ± 1.9	5.6 ± 2.3	0.09
MTV ± SD	183 ± 189	462 ± 474	0.32	10.4 ± 19.1	53.9 ± 54.6	0.18
RGD	*n* = 6	*n* = 6		*n* = 6	*n* = 6	
SUVmax ± SD	2.61 ± 1.8	3.21 ± 1.6	0.56	2.41 ± 1.1	4.43 ± 1.1	0.01
ATV ± SD	43.5 ± 67.1	21 ± 40.4	0.59	10.5 ± 8.53	96.0 ± 124	0.22

## Discussion

To the best of our knowledge, this is the first clinical study to use RGD in patients with lymphoma. Patients presenting with cHL had significantly higher RGD uptake at baseline than NHL. Our prospective study shows that non-responders had higher RGD uptake at C2 RGD compared to responders.

RGD–K5 is a product that is expensive (around 1000 €) and difficult to synthesise. Due to technical problems in production, two patients were lost, two RGD PET scans were delayed and the investigator withdrew 10 potentially eligible patients. No patients had their treatment delayed because of RGD.

An end-of-treatment RGD PET would have allowed further description of angiogenic behaviour and changes and a more thorough analysis. Unfortunately, an end-of-treatment RGD PET was not performed for many reasons: logistics, cost, radiopharmaceutical difficulties and radiation exposure with no clear therapeutic implications.

As RGD is a new tracer, there is no clear reference or validation to determine the best threshold method to calculate ATV with RGD. However, Vera et al. showed that a fixed threshold of 1.4 SUV was the best for low tumour-to-background tracers [11]. As RGD has low uptake (Table 2), we tested this method to determine RGD ATV. However, the results were disappointing. We also tested all methods described in the paper published by Vera et al., with no success [11]. Thus, we decided to determine RGD ATV by visual sampling.

Our study allowed us to measure RGD uptake in normal organs before and after chemotherapy. Chemotherapy did not affect normal organ uptake [12].

Preliminary histopathological results performed on initial biopsies showed no correlation between endothelial cells marked via the ERGmarker, the number of vascular sections and C0 and C2 RGD uptake. Endothelial cell marking via the ERGmarker is an indirect expression of neovascularisation. The number of vascular sections per 3.15 cm^2^ frame was counted. Further histopathological biopsies are necessary to confirm these results.

Patients with cHL had higher RGD uptake. cHL is mainly characterised by the presence of Reed Sternberg cells (RSCs), which are multinucleated neoplastic cells. RSCs are derived from B germinal centre and produce their own growth factors, Th2 cytokines and chemokines, creating a highly inflammatory infiltrate [13]. RSCs constitute only a minor component of the tumour (usually 1–3%); the majority of the malignancy is made up of a mixed inflammatory infiltrate variably composed of lymphocytes, eosinophils, fibroblasts, macrophages and plasma cells [14]. RSCs also express high levels of vascular endothelial growth factor (VEGF), which facilitates tumour progression [15]. The high RGD uptake in cHL compared to NHL could be due to αvβ3 overexpression in neovessels. An alternate explanation could be the presence of αvβ3 receptors on tumoural inflammatory cells. Complementary histopathological studies are being considered to verify these hypotheses.

SUVmax FDG was non-significant between responders and non-responders, because two patients presenting with DLBCL had an interim Deauville score of 4 and a final score of 2.

One of the major issues raised about RGD uptake is its ability to predict antiangiogenic drug response. Patients with DLBCL in our study had low RGD uptake. Seymour et al. [16] showed a lack of bevacizumab (anti-VEGF) efficacy in DLBCL when added to standard chemotherapy compared to chemotherapy alone. The low RGD uptake in DLBCL could therefore be linked to the lack of anti-angiogenic treatment efficiency. In contrast, patients with HL in our study had high RGD uptake. A pre-clinical study of xenografted Hodgkin lymphoma showed the efficiency of anti-angiogenic treatments [17]. No clinical data of antiangiogenic drugs in HL in humans are available. However, with the high RGD uptake in HL found in our study, the testing of anti-angiogenic treatments in refractory patients presenting with HL could be considered for further studies.

The increased C2 RGD uptake in NHL (Table 1) was not statically significant. Non-responders tended to have higher RGD uptake than responders on C2 PET (Fig. 5). Further studies are necessary to understand if the uptake is due to neoangiogenesis or an inflammatory process. Integrins are a class of heterodimeric cell surface adhesion receptors that are overexpressed on tumour endothelial cells during tumour angiogenesis, resulting in cancers that are more invasive, more migratory and better able to survive in different microenvironments. Among integrins, αvβ3 is highly expressed in tumours such as osteosarcomas, neuroblastomas, glioblastomas, malignant melanomas, and breast, lung and prostate carcinomas, but its expression is weak in most healthy organ systems (34).

The limit of our study is the small number of patients, but the patients did present with heterogeneous histological lymphoma subtypes.

## Conclusion

Our study showed a trend of higher initial RGD uptake in patients presenting with cHL compared to NHL, and non-responders also had higher post-chemotherapy RGD uptake compared to responders. Issues raised about RGD uptake, particularly in cHL, are yet to be explored and need to be confirmed in a larger population.

## Supplementary Information

**Additional file 1** Figure in additional data: RGD PET/CT (left column), PET (middle column) and CT (right column). Upper row: coronal view. Lower row: axial view. A spherical volume of interest (VOI) of diameter 3 cm is placed in the right upper lobe of the liver, avoiding the edge and any single ‘hot’ pixels likely to represent noise, sampling several axial slices to obtain a representative maximum liver SUVmax and SUVmean.

## Data Availability

The datasets used and/or analysed during the current study are available from the corresponding author upon reasonable request.

## Abbreviations

ATV: Angiogenic tumour volume
Bq: Becquerel
C0: Exam performed before chemotherapy
C2: Exam performed after 2 cycles of chemotherapy
cHL: Classical Hodgkin lymphoma
DS: Deauville score
EANM: European Association of Nuclear Medicine
ECM: Extracellular matrix
GZL: Grey zone lymphoma
MTV: Metabolic tumour volume
NHL: Non-Hodgkin lymphoma
PET: Positron emission tomography
RGD/K5 [18F]: Fluorine arginine-glycine-aspartic
