# Supplementary figures and images for: Angiogenesis imaging study using interim [18F] RGD-K5 PET/CT in patients with lymphoma undergoing chemotherapy: preliminary evidence
Source: EJNMMI Res. 2021 Apr 12;11:37. doi: 10.1186/s13550-021-00776-9 (PMC8041962; doi:10.1186/s13550-021-00776-9)

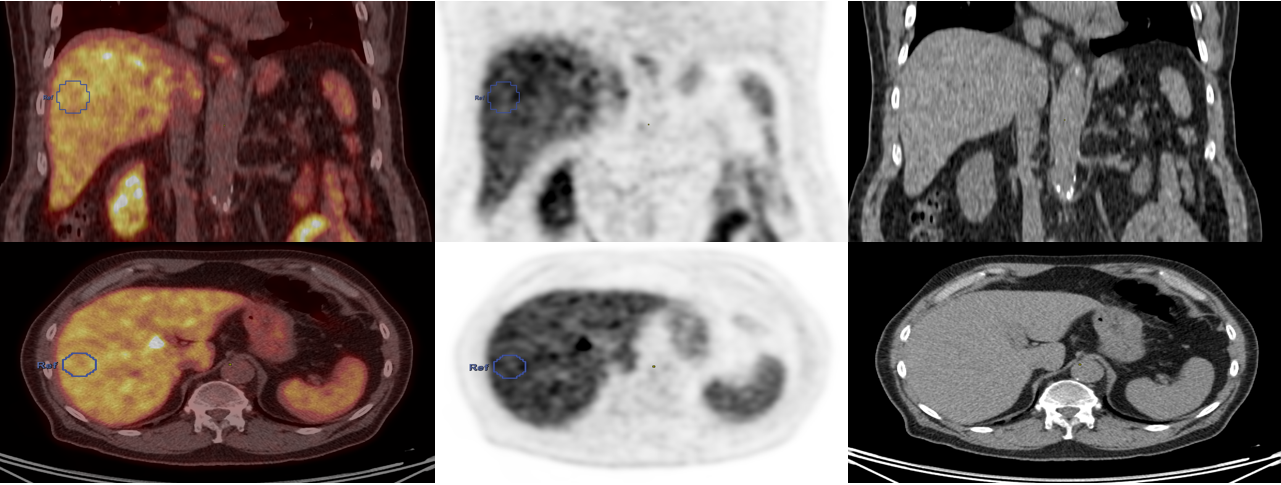

Supplement: Supplementary file 1 — Additional file 1 Figure in additional data: RGD PET/CT (left column), PET (middle column) and CT (right column). Upper row: coronal view. Lower row: axial view. A spherical volume of interest (VOI) of diameter 3 cm is placed in the right upper lobe of the liver, avoiding the edge and any single ‘hot’ pixels likely to represent noise, sampling several axial slices to obtain a representative maximum liver SUVmax and SUVmean. [file 13550_2021_776_MOESM1_ESM.docx]
